# Advances in Faba Bean Genetics and Genomics

**DOI:** 10.3389/fgene.2016.00150

**Published:** 2016-08-22

**Authors:** Donal M. O'Sullivan, Deepti Angra

**Affiliations:** School of Agriculture, Policy and Development, University of ReadingReading, UK

**Keywords:** *Vicia faba*, transcriptome, single nucleotide polymorphism, genetic linkage map, synteny, mutagenesis, transformation

## Abstract

*Vicia faba* L, is a globally important grain legume whose main centers of diversity are the Fertile Crescent and Mediterranean basin. Because of its small number (six) of exceptionally large and easily observed chromosomes it became a model species for plant cytogenetics the 70s and 80s. It is somewhat ironic therefore, that the emergence of more genomically tractable model plant species such as *Arabidopsis* and *Medicago* coincided with a marked decline in genome research on the formerly favored plant cytogenetic model. Thus, as ever higher density molecular marker coverage and dense genetic and even complete genome sequence maps of key crop and model species emerged through the 1990s and early 2000s, genetic and genome knowledge of *Vicia faba* lagged far behind other grain legumes such as soybean, common bean and pea. However, cheap sequencing technologies have stimulated the production of deep transcriptome coverage from several tissue types and numerous distinct cultivars in recent years. This has permitted the reconstruction of the faba bean meta-transcriptome and has fueled development of extensive sets of Simple Sequence Repeat and Single Nucleotide Polymorphism (SNP) markers. Genetics of faba bean stretches back to the 1930s, but it was not until 1993 that DNA markers were used to construct genetic maps. A series of Random Amplified Polymorphic DNA-based genetic studies mainly targeted at quantitative loci underlying resistance to a series of biotic and abiotic stresses were conducted during the 1990's and early 2000s. More recently, SNP-based genetic maps have permitted chromosome intervals of interest to be aligned to collinear segments of sequenced legume genomes such as the model legume *Medicago truncatula*, which in turn opens up the possibility for hypotheses on gene content, order and function to be translated from model to crop. Some examples of where knowledge of gene content and function have already been productively exploited are discussed. The bottleneck in associating genes and their functions has therefore moved from locating gene candidates to validating their function and the last part of this review covers mutagenesis and genetic transformation, two complementary routes to validating gene function and unlocking novel trait variation for the improvement of this important grain legume.

## Introduction

### Origins and importance of *Vicia faba* in world agriculture

*Vicia faba* L.(*Vf*) or faba bean is a grain legume of great importance in world agriculture due to its high yield potential compared to alternative grain legumes, its ability to fix nitrogen through symbiosis with *Rhizobium leguminosarum* in its root nodules, but most crucially for its role as a staple dietary protein source in North African and Middle Eastern cultures. The species is thought to have been domesticated in the Eastern Mediterranean region, perhaps somewhere between Afghanistan and the Eastern Mediterranean (van de Wouw et al., 2001), but no extant wild relative has yet been found although new species closely related to *Vf* have been found in recent decades (Maxted, 1993). Nonetheless, there is great variability within the domesticated genepool, with the major center of diversity centered around the Mediterranean basin and secondary centers of diversity in the Nile Valley, South America, and Central and Eastern Asia (Duc et al., 2010), providing much untapped potential to breeders. This diversity of forms is exemplified by the botanical classification of “*major*” (large-seeded or “broad” bean), “*equine*” (mid-sized or “horse” bean), and “*minor*” (small, rounded seed) types. *Vf* is one of the earliest domesticated Old World agricultural crops, with credible archaeobotanical evidence linking it to the pre-pottery Neolithic period around 10,000 BP in a site in North-West Syria (Tanno and Willcox, 2006). However, again in keeping with its great diversity and adaptability, it has a long history of utilization outside the center of origin in diverse agroecological settings from the Boreal and Atlantic Maritime climates to arid and sub-tropical regions. The most current available statistics for dry beans show it ranking 7th in terms of global production of grain legumes with production in 2013 of 1.17 million tons (FAOSTAT, 2013)^1^, though this probably grossly underestimates the nutritional and societal importance of the species as a majority of faba bean worldwide is cultivated and consumed locally by subsistence farmers. Furthermore, FAOSTAT figures relate to dry beans and there are no reliable global data for large-seeded broad bean types picked green and consumed fresh. China is the leading producer, followed currently by Ethiopia, Australia, France, Egypt, Morocco, Sudan, and the UK, though rank order amongst the latter seven countries has historically been somewhat fluid. Despite the strong cultural attachment of Mediterranean basin and Middle Eastern populations to consumption of faba bean, for a variety of reasons addressed below, many of these countries have become net importers.

### Challenges facing faba bean

#### Yield stability

Faba bean is generally speaking the most productive of grain legumes in environments where rainfall is not limiting or in irrigated conditions, and can indeed be a highly profitable crop, especially if the economic benefits of biologically fixed nitrogen and enhanced weed and disease control in subsequent crops are considered (Preissel et al., 2015). However, in common with many legumes, its yields are relatively unstable, which is thought to be one important reason underlying the low inclusion rate of leguminous crops in European agriculture in particular (Cernay et al., 2015). This inherent yield instability is thought to be due, in part, to its apparently profligate flowering habit whereby many flowers spread over many nodes are produced containing far more ovules than the capacity of the plant to fertilize and fill those potential seed sites, resulting in a high but variable rate of flower and fertilized ovule abortion. Optimal yield production in faba bean is also dependent on symbiosis with *Rhizobium leguminosarum* biovar *viciae* to produce nitrogen-fixing root nodules as well as on the pollination services of wild bee populations to ensure both optimal seed set and outcrossing rates. Pollinator insufficiency has been shown recently in the UK to explain up to 64% yield loss (Nayak et al., 2015) while a large N-fixation response to rhizobial inoculation in the field has been shown even in the presence of high natural rhizobial populations (Denton et al., 2013). On top of these variable symbiotic or mutualistic interactions are layered a host of abiotic and biotic stresses, combinations of which will exact further environment-dependent losses. The many specific pests and pathogens of faba bean include fungi (*Ascochyta fabae*—ascochyta blight, *Botrytis fabae*—chocolate spot, *Uromyces fabae*—bean rust, *Peronospora viciae* f.sp. *faba*—downy mildew), insects (*Aphis fabae*—black bean aphid, *Bruchus rufimanus*—the bruchid seed beetle), nematodes (*Ditylenchus gigas*—stem nematode), and parasitic plants (*Orobanche crenata* and *Orobanche foetida*—broomrape) to name just a few of the most prominent. Detailing the fascinating coevolutionary struggles between these diverse organisms and their common host is beyond the scope of this review and interested readers can find further details in one of many recent reviews of biotic stresses on faba bean (Pérez-de-Luque et al., 2010; Sillero et al., 2010; Stoddard et al., 2010). Chief amongst abiotic stresses are heat and drought, which play all the more important a role in yield determination because of the sensitivity of flower fertility to even mild or transient levels of stress. Fertility of developed flowers has recently been shown to be highly sensitive to heat stress, though total yield losses are mitigated by the shift of yield production to higher nodes formed after a stress event has passed (Bishop et al., 2016b). And a further study showed that bee pollination can significantly mitigate damage to fertility caused by heat stress (Bishop et al., 2016a). Cold/frost tolerance is an important trait for winter beans (Sallam et al., 2015), and tolerance to salinity soils is pertinent in semi-arid regions (Tavakkoli et al., 2012). Again, abiotic stress resistance is a wide topic which has been adequately covered in recent reviews (Khan et al., 2010; Link et al., 2010; Patrick and Stoddard, 2010).

#### Quality

Meeting the protein demand of a growing global population represents a challenge not only from the yield perspective but also from the quality perspective. As yield potential continues to increase through the efforts of breeders, it is important for the protein density of the crop to be maintained, if not improved, from typical values of around 30% dry weight. The well-established relative deficiency of faba bean in essential sulfur-containing amino acids, cysteine and methionine, can of course be balanced in food, and feed processing, but amelioration of the balance of essential amino acids would be a valuable breeding target which has not yet been addressed (Multari et al., 2015). Finally, in order for the nutritive value of faba protein to be maximized and nitrogen- and phosphorous-rich wastes in the food chain minimized, serious research is needed to identify, and remove anti-nutritional factors that inhibit the normal digestion of starch and protein in the gut (amylase and protease inhibitors), that cause oxidative stress (e.g., vicine) or that sequester key nutrients (e.g., phytic acid). Much research on faba bean quality has focussed on two key anti-nutritional factors: tannins and vicine/convicine. Vicine and convicine are particularly intriguing as the metabolized derivatives of these pyrimidine glucosides cause a serious and potentially fatal condition known as favism in genetically predisposed humans and are found only in a handful of *Vicia* spp., the most notable of which is faba bean (Ray and Georges, 2010). Seed coat tannins and vicine-convicine are naturally high in a large majority of current varieties (e.g., Khamassi et al., 2013) and have been shown to lower protein digestibility and energy content in a variety of animal feeding studies. The nutritional quality of faba bean and status of research on anti-nutritional factors have been thoroughly reviewed by Crepon et al. (2010).

Against this backdrop of high yield potential and high utility in increasing the sustainability of nitrogen cycling in agro-ecosystems and security of protein supply in the food chain, it is both timely and appropriate to review how genetic and genomics are already contributing to the accelerated breeding of high yielding, climate-resilient, and nutritious faba beans and how we can expect these technologies to be exploited in the future.

## Genomics

### Transcriptomes

The first major contribution to systematic faba bean transcriptome knowledge was the release of approximately 5000 Expressed Sequence Tags (EST) from developing embryos of a broad bean variety “Windsor” described by Ray and Georges (2010). This study provided a useful snapshot of the functional classification and relative expression level of the more abundant transcripts from the embryo transcriptome in the early to middle stages of its development, even though restricted to one genotype and one tissue. Kaur et al. (2012) and Yang et al. (2012) undertook 454 sequencing of the transcriptome specifically to underpin SSR discovery and this significantly increased the volume of transcriptome data available although in both cases from mixed genotypes. Subsequently, studies began to encompass multiple, separate inbred genotypes (Ocaña et al., 2015; Webb et al., 2016) as well as multiple tissues and genotypes (Ray et al., 2015). The deepest transcriptome coverage yet produced has come from Illumina sequencing of a library of mixed tissues enriched with embryo transfer cells from variety “Fiord” (Arun-Chinnappa and McCurdy, 2015; Zhang et al., 2015). At the time of writing, transcriptomes from nine specifically identified single genotypes and a selection of tissues including whole seedling, root, shoot, leaf, seed coat, and embryo were available (summarized in Table 1), which offers for the first time to the faba bean research community the possibility to conduct crude electronic Northern analyses and to mine genotypic variants from multiple genetic backgrounds.

**Table 1:** **Key *Vf* transcriptome datasets**.

**Bioproject**	**Genbank reference**	**Cultivar**	**Tissue**	**References**
PRJNA225873	SRP033593	BPL10	10-d seedling	Webb et al., 2016
PRJNA225881	SRP033121	Albus	10-d seedling	Webb et al., 2016
PRJNA238140	SRX476199	CDC Fatima	6-d root	Ray et al., 2015
	SRX476200	CDC Fatima	6-d shoot	Ray et al., 2015
	SRX476493	CDC Fatima	Seed coat	Ray et al., 2015
	SRX476217	SSNS-1	6-d root	Ray et al., 2015
	SRX476220	SSNS-1	6-d shoot	Ray et al., 2015
	SRX475907	A01155	6-d root	Ray et al., 2015
	SRX475873	A01155	6-d shoot	Ray et al., 2015
	SRX476566	A01155	Seed coat	Ray et al., 2015
PRJNA277609	SRP055969	Fiord	Mixed tissues	Arun-Chinnappa and McCurdy, 2015
PRJEB8906	ERP009949	Fiord	Cotyledon epidermis and parenchyma	Zhang et al., 2015
NA	JR964201- JR970413^*^	Icarus, Ascot	Mixed tissues	Kaur et al., 2012
PRJNA253768	SRP043650	NS	Leaves	Suresh et al., 2015
NA	GI:219212932 - GI:219282595	Windsor	2 week old embryo	Ray and Georges, 2010
NA	SRP045955	INRA-29H	Leaf	Ocaña et al., 2015
NA	SRP045955	*Vf*136	Leaf	Ocaña et al., 2015

NA, Not applicable; NS, Not stated.

* Only assembled contigs available as TSA.

### SNPs

The extreme paucity of *Vf* sequence of any description in public databases, lamented by previous reviewers (e.g., Gnanasambandam et al., 2012), meant that very small numbers of SNPs had been discovered prior to 2014. The first genetic linkage map of faba bean to explicitly target gene-based polymorphisms was reported by Ellwood et al. (2008), who adopted a strategy of cross-species amplification of conserved orthologs in order to identify polymorphic intron-targeted markers, which were implemented at first either as Cleaved Amplified Polymorphic Sequence (CAPS) or Single Nucleotide Primer Extension (SNuPE) assays. Later, many of these polymorphic intron sequences were converted from CAPS/SNuPE to Kompetitive Allele Specific PCR (KASP) format (Cottage et al., 2012), which made the exploitation of this first suite of SNPs more accessible. The advent of RNA-Seq datasets fueled the next wave of SNP development. Kaur et al. (2014b) designed an iSelect assay based on 768 SNPs, of which 551 were placed on a genetic map generated from a RIL population of the cross Icarus × Ascot. More recently, Webb et al. (2016) reported design of individual KASP assays for 845 SNPs mined from alignment of assembled transcriptomes of “Albus” and “BPL10” inbred lines; of these, 653 were successfully mapped. The burgeoning transcriptome datasets described in the previous section permit ever greater numbers of SNPs to be called, for e.g., Ray et al. (2015) reported 5300 unique variants where alternate alleles could be found in one more of the discovery genotypes SSNS-1, A01155, or CDC Fatima and Ocaña et al. (2015) reported 39,060 SNP and 3669 InDel polymorphisms in their analysis of transcriptomes of INRA-29H and *Vf* 136, though these larger SNP sets remain to be validated as working SNP assays.

**Figure 1:**
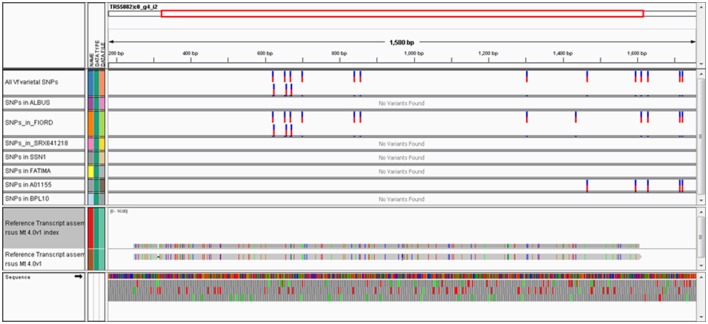
**Integrative Genomics Viewer (IGV) snapshot of SNPs in the *Vf* ortholog of Medtr2g009080 mined from public trancriptome datasets**. The 11 horizontal tracks shown in this view from top to bottom are: the meta-transcriptome contig TR55082¦c0_g4_i2 is shown as a schematic, all intravarietal SNP positions; positions where the minor (non-consensus) allele is found in each of the following genotypes: “Albus,” “Fiord,” “SRX641218,” “SSN1,” “Fatima,” “A01155,” and “BPL10,” alignment of the reference *Vf* contig TR55082¦c0_g4_i2 to the *Mt*4.0 genome (coding region of Medtr2g009080 in this instance), and in the bottom panel is shown a 3-frame translation of the *Vf* sequence. In this example, there are a total of 16 intravarietal SNPs distributed across the gene—11 unique to “Fiord,” two unique to “A001155” and three minor alleles in common to “Fiord” and “A01155,” with the remaining genotypes carrying the unchanged common allele identical to the consensus contig sequence.

The alignment of genotype-specific transcriptome datasets to well annotated references is relatively straightforward and can be accomplished using user-friendly cloud-based tools e.g., BWA-MEM for alignment (Li, 2013) and Varscan for SNP calling (Koboldt et al., 2012) implemented in the Galaxy workflow environment (Afgan et al., 2010). Figure 1 shows a typical alignment of contigs from several distinct *Vf* genotypes to a gene predicted by synteny to lie within the published VC interval (Khazaei et al., 2015). Over the 1534 bp contig, which covers the full coding region predicted in Medicago together with some 5′ and 3′-UTR sequence, there are 16 varietal *Vf* SNPs.

### EST-SSRs and genomic SSRs

The other category of molecular marker which has received much attention from the faba bean research community was Expressed Sequence Tag—Simple Sequence Repeats (EST-SSRs). As inherently highly discriminatory, co-dominant markers embedded in genic locations which can be readily associated with orthologous positions in related species, EST-SSRs are an attractive category of marker and offer the advantage that repeats can be mined from a single genetic background with relatively low sequence coverage, so were in the first wave of marker sets to be developed from transcriptome data. Initially modest numbers of EST-SSRs reported by Gong et al. (2011) and Ma et al. (2011) were followed by greater numbers of candidate and validated EST-SSRs (Kaur et al., 2012; El-Rodeny et al., 2014). Although highly portable and informative, EST-SSRs, as with all types of SSR marker, can be difficult to score in a fully automated fashion, and are inherently less amenable than SNPs and INDELs to conversion to high throughput parallel assay formats.

In contrast to EST-SSRs, genomic SSRs anchored in non-coding genomic sequence may be difficult to validate as locus-specific assays due to the prevalence of complex, repetitive DNA sequences outside coding regions, which constrain the design of locus-specific primers. Furthermore, most genomic SSRs which fall outside conserved coding regions cannot be used to make syntenic bridges to better characterized relatives. Nonetheless, genomic SSRs, mined from SSR-enriched genomic DNA libraries, have been successfully developed by Zeid et al. (2009) and Yang et al. (2012). Table 2 summarizes key molecular marker sets which have been developed to date for faba bean.

**Table 2:** **Key molecular marker sets**.

**Marker type**	**Discovery genotypes**	**Number of validated polymorphic markers**	**Number mapped**	**References**
EST-SSR	Komasake	647	552	El-Rodeny et al., 2014
Genomic SSR	Mixed	90	NA	Yang et al., 2012
EST-SSR	Icarus, Ascot	71	57	Kaur et al., 2012
SNP (KASP)	Albus, BPL10	824	687	Cottage et al., 2012; Webb et al., 2016
SNP (Illumina)	Icarus, Ascot	480	465	Kaur et al., 2014b
ITAP	*Vf*6, *Vf*27	151	127	Ellwood et al., 2008

## Genetics

Much progress has been made in the power and possibilities of genetic analysis in faba bean since the pioneering work of Adela Erith, working at the University of Reading in the 1920s, on inheritance of color, size, and form of seeds and other traits (Erith, 1930). Erith's seminal paper showed Mendelian inheritance of genes controlling readily observable (but nonetheless important) characters including hilum color, seed, and flower color and height. However, many years were to pass before the genes underlying these or any other traits have been identified. Between then and now, *Vf* genetics has passed through distinct phases which will be described below in chronological order.

### Classical genetic analysis

The period 1930–1993 was the era of molecular marker-free genetic analysis. During this period and beyond, useful foundations were laid showing simple inheritance patterns for a variety of heritable traits. As mentioned already, (Erith, 1930) found single dominant or semi-dominant genes explaining flower, seed, and hilum color as well as plant height, though there was no well-developed locus nomenclature in this early work. Faba bean received much attention in the 1960s to the early 1990s as a model for cytogenetics due to its uncommonly large haploid genome size of 13 Gb (Soltis et al., 2003) and modest haploid chromosomes number (*n* = 6), which made for large, readily observable chromosomes. For a time, the study of chromosome breakage response to a range of physical and chemical agents became quite fashionable (e.g., Menke et al., 2000; Sobita and Bhagirath, 2005; Milan and Upadhyay, 2007; Rybaczek et al., 2008) and *Vf* was one of the early crop species whose chromosomes were found to be amenable to flow sorting (Kovarova et al., 2007). The more direct relevance of cytogenetics to modern molecular genetics was the identification of asynaptic mutants (Sjödin, 1970), on which a series of trisomic stocks were founded. Genetic analyses of crosses involving trisomic parents allowed genetic markers to be assigned to physical chromosomes (Patto et al., 1999). The definitive work on pre-molecular faba bean genetics was the monumental work of Sjödin (1971) to collect, induce, classify and cross mutants and spontaneous variants in a wide variety of characters of interest. Although such comprehensive surveys of genetic variation have not since been carried out, induced and spontaneous mutants affecting a variety of traits have been described in this period. For instance, a gene controlling symbiosis, *sym-1*, was described by Duc and Picard (1986). Duc et al. (1999) reported a recessive allele of the *i1* locus conferring a green cotyledon; this has a similar phenotype as the green cotyledon allele of the *Sc* locus, mapped by Khazaei et al. (2014) to chromosome IV.

Work on genetics was not confined to visible morphological phenotypes either. Ramsay (1997) showed Mendelian inheritance of a major effect seed dormany gene, denoted *doz*, detected by scoring the timing of seed germination semi-quantitatively in a set of segregating Recombinant Inbred Lines (RILs). Unfortunately, few of the populations generated and characterized in these early genetic studies have been maintained to the present day, though the trait sources (or at least sources carrying analogous phenotypic states) have for the most part. However, quantitative traits, especially those under oligogenic control, were always going to require a molecular mapping approach so that medium or even small size effects could be placed within a sufficiently dense phenotype-independent marker framework.

### Molecular genetics

A combination of isozymes and RAPD markers formed the basis of the first *Vf* molecular marker map (Torres et al., 1993), consisting of 66 markers arranged in 11 linkage groups, in which, interestingly, the first indications of synteny between faba bean and other *Fabaceae* were noted.

From this point on, *Vf* genetic studies followed a standard pattern. Typically biparental RIL or F_2_ populations generally consisting of no more than 200 progeny lines were genotyped with non-sequence based markers, chiefly RAPD. The highlights of this phase of faba bean genetics were a series of RAPD-based QTL studies targeting resistance to pathogens and parasites—for instance, to ascochyta (Avila et al., 2004; Diaz-Ruiz et al., 2009), rust (Avila et al., 2003), and *Orobanche* (Diaz-Ruiz et al., 2010; Gutierrez et al., 2013). This early period of integration of molecular marker technology in genetic mapping studies has been reviewed by Torres et al. (2010) and Gnanasambandam et al. (2012).

In parallel with the acceleration in growth of faba bean sequence and marker datasets, there has been a correspondingly encouraging increase in the density and utility of gene-based genetic maps (Table 3). The first gene-based genetic map of *Vf*, composed in the main of 127 co-dominant, portable, Intron-Targeted Amplified Polymorphism (ITAP) markers, was that of Ellwood et al. (2008). A linkage map comprising 128 EST-SSR markers was produced by Ma et al. (2013) and a 552- locus map comprising loci generated from 235 faba bean-derived EST-SSRs and 162 markers derived by cross-amplification from red and white clover was reported recently by El-Rodeny et al. (2014). The consensus map of Satovic et al. (2013) was the first where a majority of markers mapped to just six chromosomally assigned linkage groups, though a minority of these were gene-based, transportable, co-dominant markers. More recently, 551 SNPs and 71 SSRs were combined in Kaur et al. (2014b), though not all of the 12 linkage groups reported could be definitively assigned to one of the six physical chromosomes. The densest SNP coverage available in a fully physically anchored consensus linkage map to date is that reported by Webb et al. (2016). This combined 34 SNP markers discovered in *Vf* 6/*Vf* 27 backgrounds by Ellwood et al. (2008) and converted to KASP format by Cottage et al. (2012) with 653 new “Albus” × “BPL10” SNPs into a single 687-locus consensus map with all markers mapping to just six linkage groups each of which could be assigned to a physical chromosome.

**Table 3:** **Key genetic maps**.

**Cross**	**Number of loci**	**Number of linkage groups**	**Average marker interval**	**Map length (cM)**	**References**
Consensus of six F_2_ populations	687	6	2.04 cM	1403.8	Webb et al., 2016
“Nubaria 2” × “Misr 3” F_2_	552 EST-SSR	6	1.25 cM	687.7	El-Rodeny et al., 2014
Consensus of three RIL populations	729 RAPD, ITAP, SSR, morphological	6	6.31 cM	4602	Satovic et al., 2013
91825 × K1563 F_2_	128 SSR	15	12.4 cM	1587	Ma et al., 2013
Icarus × Ascot F5:6 RILs	522 (57 EST-SSR, 465 SNP)	12	2.33 cM	1216.8	Kaur et al., 2014b
*Vf*6 × *Vf*27 RILs	127	12	13.27	1685.8	Ellwood et al., 2008; Cottage et al., 2012

### Description of synteny

The great benefit of the progress in mapping ever greater numbers of sequence-based markers is that the by now well-established conservation of gene order amongst related legume genomes could be used to anchor genetic maps from unsequenced legumes (in this instance faba bean) to the *Medicago truncatula* (*Mt*) genome. The first sequence-based genetic map of faba bean which allowed the global pattern of *Vf* -*Mt* synteny to be observed was that of Ellwood et al. (2008). Although the number of markers (127) was modest, a clear picture of extensive macrosynteny emerged. El-Rodeny et al. (2014) and Kaur et al. (2014b) elaborated this picture with a greater marker density, but the clearest and most complete picture of the extent of macrosynteny between *Vf* and *Mt* comes from the Webb et al. (2016) study. All 653 newly discovered and mapped SNP markers in this latter study were selected following highly conservative filtering for single copy *Vf* sequences with a clear best reciprocal BLAST hit relationship with a single copy gene in *M. truncatula*. Reflecting these stringent marker design criteria, the name of the each new (Webb et al., 2016) *Vf* marker carries explicit reference to its presumed *Mt* ortholog. Combined with the fact that each linkage group in the Webb et al. (2016) consensus map corresponds to a full physical *Vf* chromosomes meant that for the first time all six *Vf* chromosomes could be aligned to the *Mt* sequence without spurious interruptions to macrosynteny pattern caused by lack of marker coverage.

### Exploitation of synteny

The hallmark of “pre-synteny” genetic studies in all species, *Vf* being no exception, was that the reporting of a given gene or QTL in proximity of a certain molecular marker was quite frequently the end of the story as there was no method to target markers to a region of interest without screening vast numbers of anonymous markers such as AFLPs or RAPDs.

The year 2012 marked a new departure for faba bean with sequence-based and synteny-anchored marker maps being applied to flowering time QTL (Cruz-Izquierdo et al., 2012). Likewise, detection of ascochyta resistance QTL by Kaur et al. (2014b) permitted localization of QTL in regions with clear colinearity to fully sequenced model genomes. Khazaei et al. (2014) studying stomatal traits and Khazaei et al. (2015) mapping vicine-convicine content were able to do likewise. In theory now, a given QTL would be associated with an interval whose gene content and even order could be predicted by exploiting synteny with Medicago and marker development efficiently targeted to this predicted gene content.

As outlined in the previous sections, much of this progress has stemmed from the exploitation of sequencing and cost-effective genotyping technologies to achieve reasonably dense coverage of all six *Vf* chromosomes with sequence-based molecular markers that allow the *Vf* gene-based genetic map to be confidently aligned in large part with fully sequenced reference genomes such as Medicago (Young et al., 2011) or soybean (Schmutz et al., 2010).

**Figure 2:**
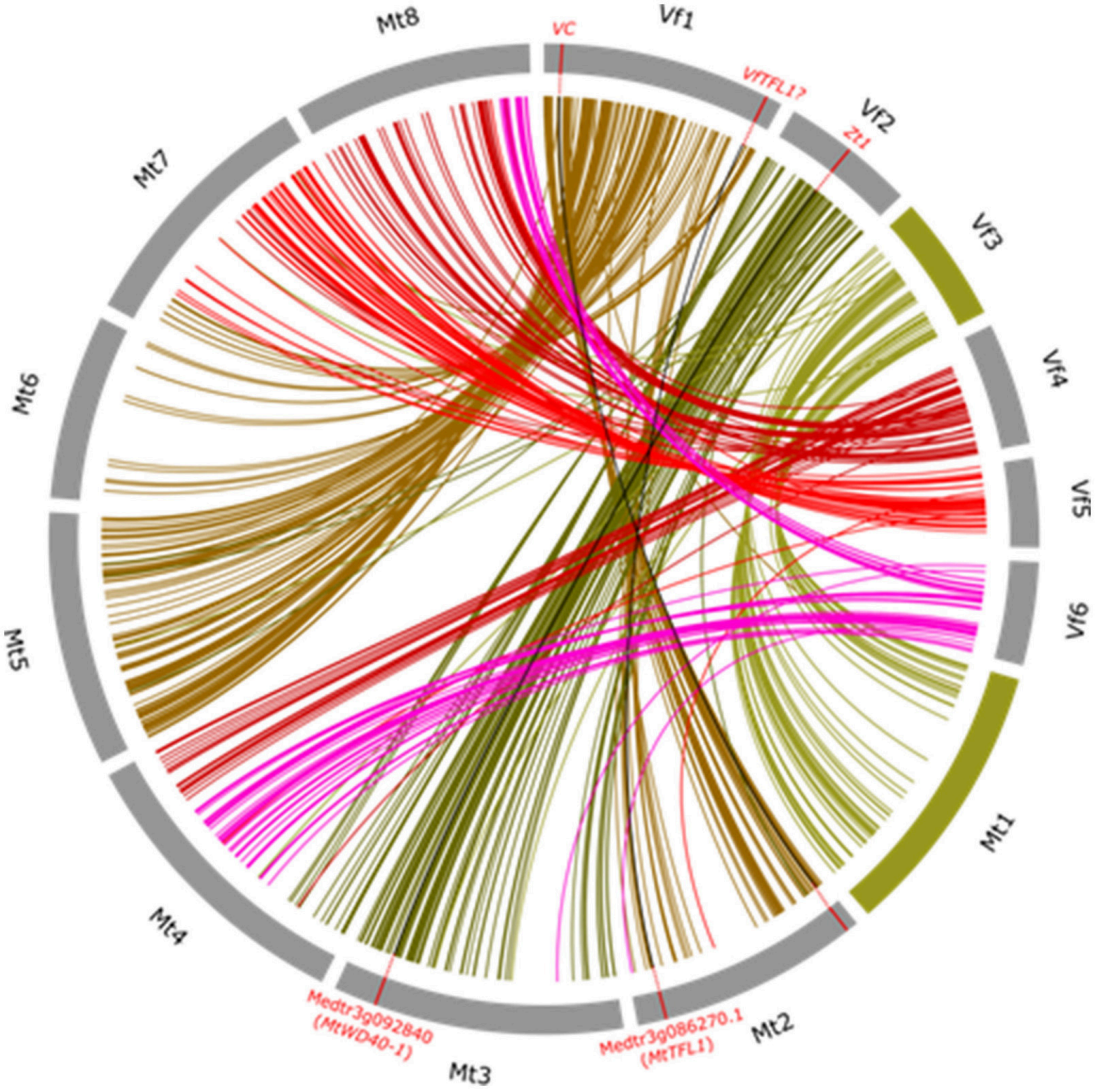
**Wheel representation of synteny between pseudomolecule sequence of Medicago truncatula chromosomes *Mt*1-*Mt*8 and the consensus *Vf* linkage map (*Vf*1–*Vf*6)**. Syntenic relationships were as described in Webb et al. (2016) and re-drawn for this figure using “Circos” software (Krzywinski et al., 2009). Link lines to Medicago orthologs for markers on each *Vf* chromosome are shown in a different color. The virtually uninterrupted collinearity between the whole of *Vf* 3 and the whole of *Mt*1 is highlighted by representing these orthologous chromosomes in green. To illustrate how useful this syntenic framework is for translational genomics as well as for drawing together knowledge of the genome, three exemplar loci where there is a proven or presumed orthologous relationship are shown as red ticks on the orthologous chromosomes and linked by black lines. These are: the mapped *ZT1* locus and its Medicago ortholog *MtWD40-1*, the mapped genetic interval corresponding to the mapped *VC* (vicine-convicine) locus and its corresponding *Medicago* syntenic interval on *Mt*2, and the actual position of the *Medicago Terminal Flower Locus 1* (*TFL1*) with a link drawn to the region of *Vf* 1 where it is presumed the *Vf* ortholog (*VfTFL1*) is likely to be located.

The exploitation of macrosynteny between crop and model genomes has a number of benefits. Firstly, once a trait has been mapped to a genetic interval which aligns well to a segment of a model sequenced genome, knowledge of gene function in the model species can be translated back to target crop species. In an instance illustrated in Figure 2, *ZT1* (controlling flower pigmentation as well as seed coat tannins) was mapped to the *Vf* _Mt3g092810_001–*Vf* _Mt3g094760_001 interval, with clear synteny to a portion of Mt3. Perusal of the annotation information for the syntenic interval in Medicago revealed a logical biological candidate in the form of the *Transparent Testa Glabra 1* (*TTG1*) WD40 transcription factor (Medtr3g092840), which had previously been shown in *Mt* to determine flower color, and in the follow-up of this hypothesis, a deletion in the recessive allele sequence was found to plausibly explain the *zt* (unpigmented) phenotype (Webb et al., 2016). This co-called translational genomics approach leverages prior investment in model species biology and fast-tracks causative allele identification. Secondly, quite independently of translation of biological information, molecular marker targeting to specific regions of interest is possible using the syntenic framework. This is relevant in situations where the target interval is large or initial candidate functional information is absent and is illustrated in Figure 2 by the case of SNP mining targeting the *VC* locus. The published interval in which this gene maps is collinear with part of Mt2 (Khazaei et al., 2015) and SNPs discovered in sequences orthologous to Medicago genes in the syntenic interval can be used selectively and cost-effectively in further high-resolution mapping of the locus. Thirdly, this syntenic framework can in principle be used to reverse map genes in *Vf*. Here, we use the example of the *Vf TERMINAL FLOWER 1* (*VfTFL1*) gene. Avila et al. (2007) examined the translational hypothesis that an ortholog of the *TFL1* gene controls determinacy of flowering in *Vf* as it does in *Arabidopsis*, soybean and numerous other legume and non-legume species by showing correlation of the determinate type with a diagnostic non-synonymous substitution in a conserved residue of the coding region across a diverse panel of determinate and indeterminate types. They did not, however, genetically map *VfTFL1*. Once again, clear macro-colinearity between the *Vf* region corresponding to the *Mt* region harboring *MtTFL1* suggests a working hypothesis (which remains to be proven) that the published *VfTFL1* sequence should map to the long arm of *Vf* chromosome 1.

## Genomic diversity

Another arena in which genomics is playing a crucial role is in the description and rational exploitation of diverse, but often poorly characterized, genetic resources. Despite the lack of a known wild progenitor species, the domesticated *Vf* genepool, as alluded to in the introduction, is extremely diverse. This diversity is manifest in the plethora of morphological forms and adaptations to diverse agroecological settings. However, full exploitation of this diversity for faba bean improvement necessitates efficient methods for quantifying and mapping genomic diversity. In parallel with the development of SSAP, AFLP, SSR, and SNP marker types, a succession of studies have used increasingly powerful molecular marker sets to quantify and map genomic diversity.

One of the first surveys of genomic diversity in diverse *Vf* germplasm was carried out by Sanz et al. (2007) using retrotransposon-based Sequence-Specific Amplified Polymorphism (SSAP) markers. This study found relatively little fine genetic structure and the first molecular evidence that “*major*,” “*minor*,” and “*equine*” lines do not form distinct clades, but rather are completely dispersed across the *Vf* phylogeny. Due to the low numbers of *Vf* lines (*n* = 20), it was not possible to draw any conclusions on geographic partitioning of diversity, though it was noted that samples from the better-represented countries in the study were well dispersed across the phylogenetic tree. These themes were recapitulated by Zeid et al. (2003) using AFLP markers to genotype 79 inbred lines from Europe, North Africa and Asia. Again, “*major*” and “*minor*” botanical types were clearly shown not to be genetically distinct, though here for the first time was a suggestion that the (*n* = 8) lines of Asian origin formed a genetically differentiated group. The AFLP-based study of Zong et al. (2009) took this further with a study of large numbers of Chinese landraces (*n* = 204) from the Chinese Academy of Agricultural Sciences (CAAS) germplasm collection, mainly winter types. They found the mainland Chinese winter germplasm to be completely distinct from other Asian, African and European diverse lines included for comparison, in line with the long history of cultivation of winter-type faba bean in relatively isolated mountainous regions of China. Kwon et al. (2010), using Targeted Region Amplified Polymorphism (TRAP) markers, also observed Chinese landraces drawn from the United States Department of Agriculture (USDA) germplasm collection (*n* = 107) to cluster completely separately from 30 comparator lines from Asia and Europe. However, the largest study of faba bean genomic diversity to date has been reported by Wang et al. (2012a), whose large sample (*n* = 802) confirmed not only the genetic distinctness of Chinese germplasm from African, European, and other Asian germplasm, but for the first time, convincingly showed differentiation amongst Chinese provinces and between winter and spring ecotypes. SNP genotyping platforms have been more recently used on small samples of diversity (Cottage et al., 2012; Kaur et al., 2014a) and the prospect of wider deployment of SNP markers on larger panels of diverse material promises the possible foundations for future *Vf* genome-wide association studies.

## Functional genomics

### Mutagenesis

The potential of mutagenesis as a tool for breeding was amply demonstrated by Sjödin (1971), reviewing not only observations made in the extensive mutagenesis programme run by Swedish breeders Svalov-Weibull in the “Primus” genetic background, but also a series of spontaneous mutants reported by a host of previous researchers. The only other mutageneis programme reported in the literature was 23 years later, when the isolation of five nodulation mutants in a screen of 20,000 M_2_ EMS-mutagenized lines in the cv. “Ascott” background was reported by Duc (1995).

**Figure 3:**
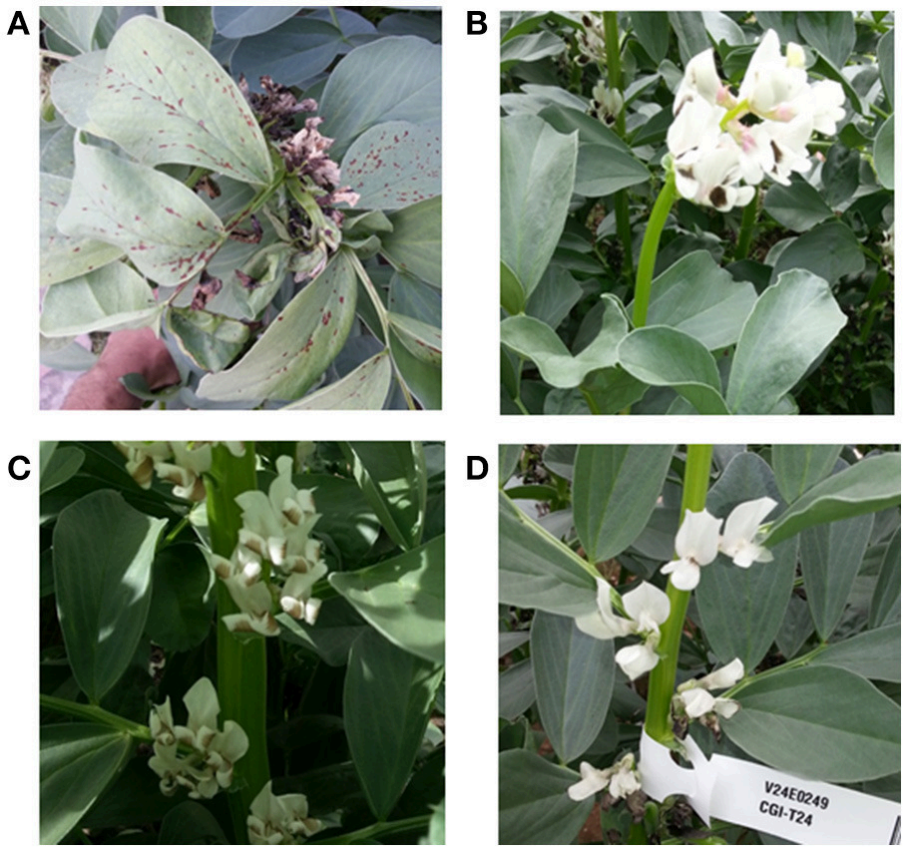
**Mutant phenotypes observed in X-ray and EMS M_2_ lines grown at the University of Reading in 2015. (A)** disease lesion mimic, **(B)** determinate flowering, **(B)** curled wing petal, **(C)** disease lesion mimic, **(D)** reduced flower pigmentation.

Interest in mutagenesis has undergone something of a resurgence in recent years. For example, the control of the devastating parasitic weed, *Orobanche crenata*, referred to in an earlier section, could be effectively controlled, together with a range of other troublesome broad-leaved weeds, if faba bean varieties with target-site mutations that render them insensitive to particular actives were developed (Gressel, 2009). Examples are the Ser653 and Ala205 mutations in the AcetoLactate Synthase target of imidazolinone and amidosulfuron families of herbicide actives, which do not occur in nature, but have been documented to occur at low frequency under strong selection pressure in the field, and there are examples in numerous crop species of targeted isolation of induced mutants (reviewed in Tan et al., 2005). At the time of writing, a number of imazapyr resistance mutations have been identified by Mao et al. (2014) and are undergoing further characterization. As a further example of the ongoing mutagenesis programmes, Figure 3 shows a selection of phenotypes observed in ongoing M_2_ X-ray and EMS populations grown during 2015 at the University of Reading.

Whilst the primary driver for contemporary mutagenesis programmes remains the identification of novel induced variants for direct incorporation in active breeding programmes, a broader motivation can be ascribed to some of the recent activity. Until efficient genetic transformation systems have been adopted and made available as a service, reverse genetics in the form of TILLing (Till et al., 2003) may be one of the most amenable techniques for validation of candidate genes identified via synteny-based approaches, especially if combined with powerful re-sequencing techniques to identify the causative mutations in a single step (Wang et al., 2012b).

### Genetic transformation/genome editing

Genetic modification represents both a research tool, permitting testing of hypotheses on gene function by over-, mis-expression or knockdown/knockout studies and an outlet for genetic research in generation of targeted phenotypic modifications based on knowledge of gene function. Stable germline transformation of *Vf* using *in vitro* regeneration of *Agrobacterium*-infiltrated (non-meristematic) internode stem segments was first reported by Böttinger et al. (2001). Adopting a somewhat different strategy, Hanafy et al. (2005) infiltrated excised (meristematic) embryo axes with *Agrobacterium* and successfully recovered stable transgenic lines. Both methods, however, reported low primary transformation efficiencies and relied on micro-grafting of putative transgenic shoot material onto non-transgenic roots, a slow and highly manual process. Hanafy et al. (2013) later reported abiotic stress resistance phenotypes of *Vf* transgenic lines overexpressing potato PR10a using their previous methods. This remains to our knowledge the sole successful demonstration to date of the feasibility of a biotechnological approach to *Vf* improvement. In the absence of a robust and efficient transformation method, some attention has been devoted to the task of decreasing generation time using tissue-culture based embryo rescue, with some success (Mobini et al., 2015). However, the search for an efficient transformation method has been recently taken up by other groups (e.g., Abdelwahd et al., 2014).

The prospects afforded by new insights into the phenotypic effects of allelic variation and the more refined biotechnological possibilities afforded by rapidly maturing genome editing technologies (Gaj et al., 2013) could potentially stimulate renewed interest in genetic transformation. An example of a game-changing product which could readily be generated using even a medium efficiency transformation system would be herbicide resistance obtained by directed mutagenesis of endogenous herbicide target genes e.g., introduction of heterologous glyphosate resistance of bacterial origin.

## Conclusions

*Vicia faba* genetics and genomics is now in a much healthier state than it was just a few years ago. We can take heart from the accelerating progress in gene identification and in production of outputs relevant to contemporary methods of breeding. We have seen the transformative effects of transcriptome re-sequencing in opening the doors to high density gene-based marker discovery and mapping. In the near future, we expect that a high throughput SNP chip incorporating many 10's of thousands of genome-wide SNPs discovered from a wide variety of genetic backgrounds will be produced and made available for community use, as well as giving rise to an ultra-high density SNP map in which perhaps as much as half of all *Vf* genes are genetically mapped. In parallel, we should see progress in the application of genotype-by-sequencing methods, both for the unbiased assessment of genetic relationships, breeding applications such as genomic selection and in trait mapping approaches based on bulked segregant analysis. Faba bean's modest but well-linked community of researchers would do well however, to set its sights on a series of even more ambitious targets to enable the community as a whole to elevate its work onto a higher plane of achievement and impact. For example, given the many highly cost effective sequencing and assembly technologies now available, a comprehensive genomic scaffold and haplotype map is surely now within reach. The history of modern agricultural genomics shows the transformative effects of a well-annotated reference genome. Communities of crop researchers who have organized themselves and published strategic roadmaps requiring centralized investment in a professional genome assembly and annotation have captured significant R&D investment and transformed the profile and fate of their communities (rice, wheat, soybean, *Phaseolus*, cowpea). Similarly, on functional genomics platforms: whilst there are no doubt training benefits to having numerous small mutagenesis programmes dotted around the globe, it could be argued that the global community needs one well-funded programme scoped to guarantee saturation mutagenesis, to catalog the mutations obtained in a public sequence database and to distribute mutant seed to research groups freely on request, an unlikely outcome from nationally funded programmes. Likewise, transformation and genome editing technologies could advantageously be developed and provided as an efficient service from a centralized laboratory.

## Author contributions

DO drafted the manuscript and provided original Figures 1, 3. DA provided information for the section on Genomics and the original Figure 2. DO and DA edited, reviewed and approved the final version of the manuscript.

## Funding

Work on genetic resources of faba bean in DOS laboratory is part-funded by the Pulse Crop Genetic Improvement Network (CH0103) project funded by the UK Department for Environment, Food and Rural Affairs (Defra).

### Conflict of interest statement

The authors declare that the research was conducted in the absence of any commercial or financial relationships that could be construed as a potential conflict of interest.

## Footnotes

^1^FAOSTAT (2013). Available online at: http://faostat3.fao.org/home/E (accessed on 10 December 2015).
